# LysM Receptor-Like Kinase LYK9 of *Pisum Sativum* L. May Regulate Plant Responses to Chitooligosaccharides Differing in Structure

**DOI:** 10.3390/ijms22020711

**Published:** 2021-01-12

**Authors:** Irina V. Leppyanen, Olga A. Pavlova, Maria A. Vashurina, Andrey D. Bovin, Alexandra V. Dolgikh, Oksana Y. Shtark, Igor V. Sendersky, Vyacheslav V. Dolgikh, Igor A. Tikhonovich, Elena A. Dolgikh

**Affiliations:** 1 All-Russia Research Institute for Agricultural Microbiology, Podbelsky chausse 3, 196608 Saint Petersburg, Russia; irina_leppyanen@mail.ru (I.V.L.); dobbi85@list.ru (O.A.P.); avrora5901@mail.ru (M.A.V.); andy-piter2007@mail.ru (A.D.B.); sqshadol@gmail.com (A.V.D.); oshtark@yandex.ru (O.Y.S.); arriam2008@yandex.ru (I.A.T.); 2 All-Russia Research Institute for Plant Protection, Podbelsky chausse 3, 196608 Saint Petersburg, Russia; senderskiy@mail.ru (I.V.S.); dol1slav@yahoo.com (V.V.D.)

**Keywords:** *Pisum sativum* L., lysin-motif receptor-like kinase *Ps*LYK9, *lyk9* mutants, AM symbiosis, phytopathogenic fungi, heterologous synthesis, co-immunoprecipitation, binding, microscale thermophoresis

## Abstract

This study focused on the interactions of pea (*Pisum sativum* L.) plants with phytopathogenic and beneficial fungi. Here, we examined whether the lysin-motif (LysM) receptor-like kinase *Ps*LYK9 is directly involved in the perception of long- and short-chain chitooligosaccharides (COs) released after hydrolysis of the cell walls of phytopathogenic fungi and identified in arbuscular mycorrhizal (AM) fungal exudates. The identification and analysis of pea mutants impaired in the *lyk9* gene confirmed the involvement of *Ps*LYK9 in symbiosis development with AM fungi. Additionally, *Ps*LYK9 regulated the immune response and resistance to phytopathogenic fungi, suggesting its bifunctional role. The existence of co-receptors may provide explanations for the potential dual role of *Ps*LYK9 in the regulation of interactions with pathogenic and AM fungi. Co-immunoprecipitation assay revealed that *Ps*LYK9 and two proposed co-receptors, *Ps*LYR4 and *Ps*LYR3, can form complexes. Analysis of binding capacity showed that *Ps*LYK9 and *Ps*LYR4, synthesized as extracellular domains in insect cells, were able to bind the deacetylated (DA) oligomers CO5-DA–CO8-DA. Our results suggest that the receptor complex consisting of *Ps*LYK9 and *Ps*LYR4 can trigger a signal pathway that stimulates the immune response in peas. However, *Ps*LYR3 seems not to be involved in the perception of CO4-5, as a possible co-receptor of *Ps*LYK9.

## 1. Introduction

To develop new approaches to regulating plant resistance or susceptibility to various microorganisms, their influence onto plants needs to be comprehensively investigated. Resistance to pathogens is determined by the plant’s capacity to recognize the surface components or secreted molecules of microorganisms, such as fungal chitin and chitooligosaccharides (COs), bacterial peptidoglycans, flagellins, the Tu elongation factor (EF-Tu), lipopolysaccharides (LPSs) and others. Such molecules are so-called microbe-associated molecular patterns (MAMPs) that are perceived by pattern recognition receptors (PRRs) [1,2]. The most well-studied MAMP is the bacterial peptide flg22, a conserved flagellin epitope with elicitor activity, which is recognized by the leucine-rich repeat receptor-like kinase (LRR-RLK) Flagellin-Sensitive 2 (FLS2) in *Arabidopsis* and rice [3,4,5,6]. The peptides elf18 and elf26, which correspond to the N-terminal end of EF-Tu, trigger immune responses [7]. These peptides are recognized by another LRR-RLK, such as the EFR1 (EF-Tu Receptor 1) in *Arabidopsis* [8,9,10]. Many phytopathogenic fungi contain chitin and chitosan in the cell wall, which are degraded by plant enzymes, causing a mixture of low molecular weight COs to be released into the medium. Compounds that have a degree of polymerization of six or more were shown to induce strong defense reactions in plants, such as the production of reactive oxygen species (ROS), defense gene expression, synthesis of phytoalexins and others. In contrast, the structurally related compounds such as short-chain CO4–5 and lipo-chitooligosaccharide (LCO) signals’ Myc factors produced by arbuscular mycorrhizal (AM) fungi play an essential role in symbiosis development. Furthermore, it was recently shown that treatment of plants with long-chain COs (such as CO6–8) may stimulate not only defense, but also symbiotic reactions, including Ca-spiking and symbiotic gene expression, via the signal pathway involved in symbiosis regulation [11]. Moreover, the short-chain CO4–5 may also stimulate the defense reactions in plants, albeit at an insignificant level [11]. This finding demonstrates that the mechanisms by which the plants can distinguish such structurally related compounds are still not well understood. How these compounds are perceived by plants requires further investigation.

Among the plant PRRs, a special class of lysine motif receptor-like kinases (LysM-RLKs) are involved in the recognition of compounds consisting of N-acetyl-D-glucosamine (GlcNAc) residues. These LysM-RLKs play an important role in the recognition of compounds stimulating defense reactions in plants, such as chitin, peptidoglycan, and their derivatives, and compounds stimulating symbiosis development, including Nod factors, Myc factors and CO4-5 [12,13,14,15,16]. Indeed, several such receptors, which are predominantly involved in the perception of chitin, peptidoglycan, and their derivatives, have been reported in various plant species. In the model plants *Arabidopsis thaliana* and rice *Oryza sativa*, the LysM-RLKs *At*CERK1 and *Os*CERK1 have been identified. To recognize the peptidoglycan, trimeric complexes such as *At*CERK1 and the LysM receptor-like proteins (LysM-RLPs) *At*LYM1 and *At*LYM3, as well as *Os*CERK1 and the LysM-RLPs *Os*LYP4 and *Os*LYP6, are formed [17,18]. The homo- and hetero-oligomeric complexes of *At*CERK1 (*At*CERK1/*At*CERK1) and LysM-RLKs with the non-active kinase domain *At*LYK5 (*At*CERK1/*At*LYK5) as well as *Os*CERK1 with LysM-RLP *Os*CEBiP are involved in the recognition of chitin and its derivatives [17,19,20,21,22,23]. *Os*CEBiP is the major long-chain CO receptor in rice, whereas *Os*CERK1 does not appear to bind COs directly [19,24,25]. However, the *Os*CERK1 from rice may play a bifunctional role, and in complex with the co-receptor *Os*LYK2, stimulates the recognition of CO4-5 and activation of specific symbiotic reactions [15,26,27]. Similarly, a bifunctional role of the homolog of the CERK1 receptor in the banana *Musa acuminata* (*Ma*LYK1) during pathogenic and symbiotic interactions has been identified [28]. Therefore, the *Os*CERK1 or *Ma*LYK1 may be a component of different receptor complexes that trigger symbiosis or defense.

Recently, the closest homologs of CERK1 have been identified in legumes: *Lj*LYS6 from *Lotus japonicus, Mt*LYK9 from *Medicago truncatula* and *Ps*LYK9 from *Pisum sativum* [14,16]. Additionally, in *L. japonicus* and *M. truncatula,* the co-receptors *Lj*LYR4 and *Mt*LYR4 were shown to form complexes with the *Lj*LYS6 and *Mt*LYK9 respectively, and bind chitin and long-chain COs [11,14]. Analysis of mutants impaired in the *lyk9* gene in *M. truncatula* showed that this receptor is also important for AM symbiosis development [11,29]. Moreover, *Mt*LYK9 binds with CO8 and has recently been found to bind with CO4-5 [11,14]. The existence of co-receptors may explain the dual role of CERK1-like receptors in the regulation of interactions with pathogenic and AM fungi in legume plants. However, the co-receptors important for recognition of CO4-5 have not yet been identified.

Here, several pea mutants impaired in the *lyk9* gene were identified, enabling us to perform their phenotypic analysis. It allowed us to verify the role of pea LysM-RLK *Ps*LYK9 in interactions with phytopathogenic fungi and AM fungi. Moreover, we examined the binding capacity of the receptor *Ps*LYK9 with different COs and its interaction with potential co-receptors in our experiments.

## 2. Results

### 2.1. Identification of Pea Lyk9 Mutant Lines in Targeting-Induced Local Lesions in Genomes (TILLING) Collection

Previously, we reported the identification of LysM receptor-like kinase (LysM-RLK) *Ps*LYK9 in the pea (*P. sativum* L.) [16]. Using an RNA interference approach, the importance of the LysM-RLK *Ps*LYK9 for the regulation of plant resistance to phytopathogenic fungi was shown. Based on the analysis of the marker genes, induced after the treatment of pea plants with CO5 and specific for symbiosis development with AM fungi, the involvement of *Ps*LYK9 in the control of this type of symbiosis was predicted. In the current study, a screening of the pea TILLING collection was performed and resulted in the identification of mutants impaired in the *lyk9* gene. This allowed us to verify the role of pea LysM-RLK *Ps*LYK9 in interactions not only with phytopathogenic fungi but also AM fungi.

Searching for mutants in the *P. sativum Lyk9* gene was conducted using the TILLING platform [30]. Preliminary screening revealed that six mutant lines were predicted to be disrupted in *Ps*LYK9 protein function (according to in silico analysis with the Sorting Intolerant From Tolerant (SIFT) program (https://sift.bii.a-star.edu.sg/); Supplementary Materials Table S1). Three mutant lines with *Lyk9* gene homozygosity (M4 and M5 generations) were chosen for the analysis and characterized in detail. Among these, the *lyk9-1* mutant line had mutations that resulted in G485R replacement in the kinase domain (in the activation loop nearby the YAQ motif), the *lyk9-2* mutant line had a mutation that resulted in L470F replacement in the kinase domain (in the activation loop) and the mutation in the *lyk9-3* line led to P145L replacement in the LysM2 motif of the extracellular domain (ECD) (Table 1). These mutant lines were used for future experiments.

### 2.2. Analysis of Symbiosis Development with AM Fungi R. irregularis in Lyk9 Mutant Lines

**Figure 1:**
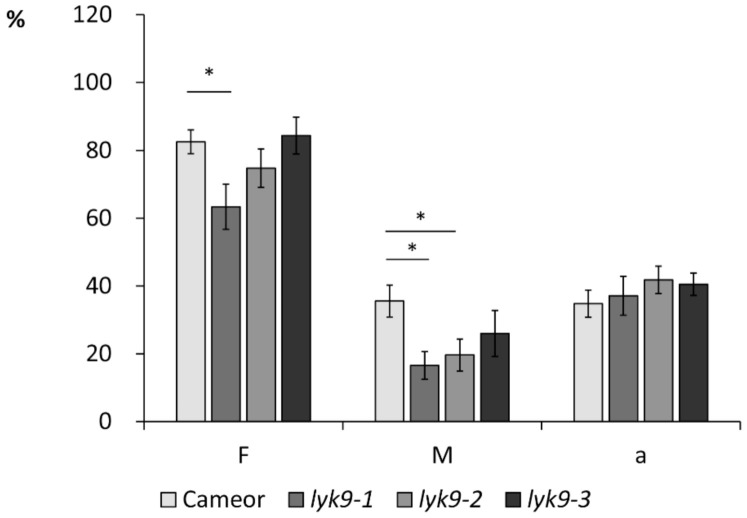
Comparative analysis of root colonization by *Rhizophagus irregularis* DAOM 197198 in the *P. sativum lyk9-1*, *lyk9-2*, *lyk9-3* mutants and their parental cultivar Cameor. *F*%—incidence of mycorrhizal infection (colonization), *M*%—intensity of mycorrhizal (internal) colonization of the root system, *a*%—arbuscule abundance in mycorrhizal root fragments. The asterisks indicate statistically significant differences based on one-way analysis of variance (one-way ANOVA), followed by Tukey post-hoc test (* *p* < 0.05).

To assess the possibility of LysM-RLK *Ps*LYK9 involvement in AM symbiosis development, we infected three mutant lines, *lyk9-1*, *lyk9-2*, and *lyk9-3*, with *R. irregularis* DAOM 197198, and estimated the parameters of mycorrhization 4 weeks after infection. The analysis revealed effective colonization with AM fungi in wild-type cv. Cameor plants. In contrast, *lyk9-1* and *lyk9-2* mutant lines showed lower levels of colonization by mycorrhizal fungi than inoculated wild-type cultivar Cameor plants for the intensity (*M%*) of mycorrhiza in the whole root system (Figure 1). In case of *lyk9-1,* we also observed the statistically significant decrease in the frequency (*F*%) of mycorrhiza in the roots. No significant changes in mycorrhizal colonization of *lyk9-3* mutant roots were observed, probably owing to the weak impacts of this mutation on *Ps*LYK9 protein function. The decreased frequency of *R. irregularis* in root fragments (*F%*) and intensity of intraradical mycelium development (*M%*) in mutants *lyk9-1* and *lyk9-2* indicate the importance of LysM-RLK *Ps*LYK9 for the control of the early stages of symbiosis development in peas.

### 2.3. Analysis of the Symbiotic and Defense Marker Gene Expression in Wild-Type Pea Plants and Mutants Impaired in the Lyk9 Gene after CO5 and CO8-DA Treatment

**Figure 2:**
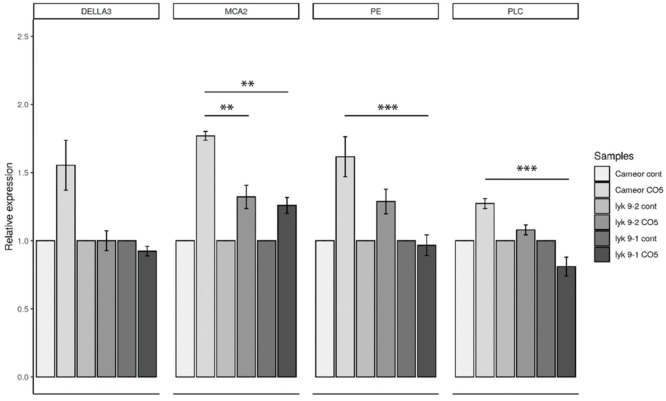
Expression pattern of *DELLA3, MCA2, PE* and *PLC* genes in pea roots treated with 10^−5^ M CO5 for 24 h. As a control, medium-treated plants were used. The expression was normalized against the constitutively expressed ubiquitin and actin genes. For each gene, the transcript level in non-inoculated roots of wild-type or mutants was set to 1 (control), and the level in treated roots was calculated relative to the control values. The graphs show the results of three independent experiments. The error bars represent standard errors of the mean (SEM) of three repeats. The asterisks indicate statistically significant differences based on one-way analysis of variance (one-way ANOVA), followed by Tukey’s post-hoc test results. (** *p* < 0.01, *** *p* < 0.001).

We previously suggested that the LysM-RLK *Ps*LYK9 may be involved in the recognition of COs with differing degrees of polymerization and acetylation in pea plants [16]. To verify this, marker gene expression was analyzed in wild-type and *lyk9* mutant lines treated with various COs. The activation of specific markers of AM symbiosis development in response to exogenously applied CO5 was estimated in wild-type plants and *lyk9-1* and *lyk9-2* mutant lines. After 24 h of treatment, the expression levels of a number of marker genes were lower in *lyk9-1* and *lyk9-2* mutants than in cv. Cameor plants (Figure 2). After 24 h of treatment with CO5, the *DELLA3*, *MCA2*, *PE* and *PLC* gene expression levels were lower in *lyk9-1* and *lyk9-2* mutants than in treated cv. Cameor plants.

**Figure 3:**
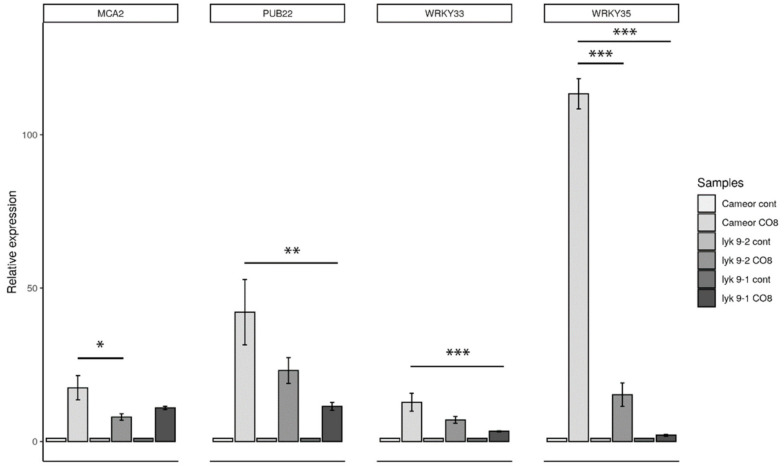
Transcript levels of *MCA2, PUB22, WRKY33* and *WRKY35* defense response genes in pea roots treated with 10^−5^ M CO8-DA for 24 h. As a control, medium-treated plants were used. Relative expression was normalized against constitutively expressed ubiquitin and actin genes. For each gene, the transcript level in non-inoculated roots of wild-type or mutants was set to 1 (control), and that in treated roots was calculated relative to the control values. The graphs show the results of three independent experiments. The error bars represent SEM of three repeats. For one biological repeat, the fragments of non-inoculated main roots or nodules from 3–4 plants were collected and used to isolate RNA. The asterisks indicate statistically significant differences based on one-way analysis of variance (one-way ANOVA), followed by Tukey’s post-hoc test results. (* *p* < 0.05; ** *p* < 0.01, *** *p* < 0.001).

Furthermore, we analyzed the expression levels of defense marker genes in the roots of *lyk9-1* and *lyk9-2* mutants in response to CO8-DA treatment (Figure 3). Both mutants had significantly lower expression levels of *MCA2*, *PUB22*, *WRKY33* and *WRKY35* than treated wild-type cv. Cameor plants. Therefore, LysM-RLK *Ps*LYK9 may regulate pea plant responses to COs differing in the degree of polymerization and acetylation, which is manifested in the decreased expression levels of marker genes.

### 2.4. Synthesis of the PsLYK9-ECD, PsLYR4-ECD and PsLYR3-ECD Extracellular Domains (ECDs) in E. coli and Co-Immunoprecipitation Assay

**Figure 4:**
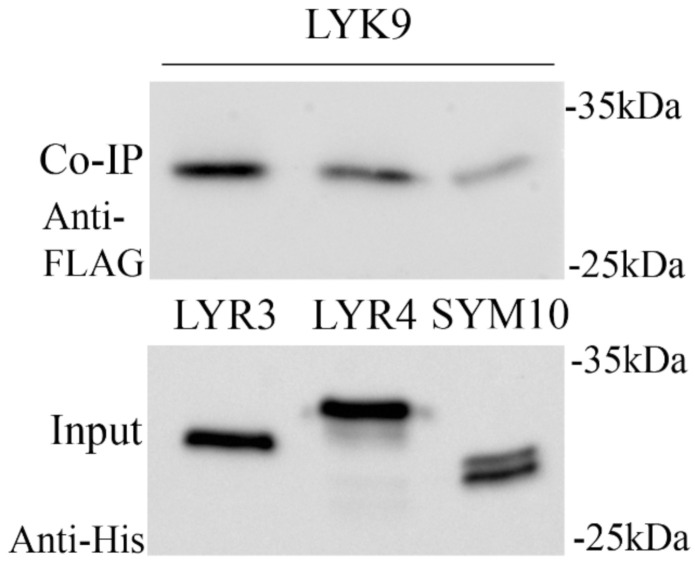
Analysis of complex formation between *Ps*LYK9-ECD tagged with FLAG and *Ps*LYR3-ECD, *Ps*LYR4-ECD, or *Ps*SYM10-ECD tagged with HIS determined by a co-immunoprecipitation assay. Extracellular domains of *Ps*LYK9, *Ps*LYR3, *Ps*LYR4, or *Ps*SYM10 were synthesized in *E. coli*. After extraction and incubation of the proteins on ice for 1 h, proteins were subjected to immunoprecipitation with anti-HIS resin and analyzed using immunoblotting with anti-FLAG and anti-HIS.

*Ps*LYK9 may be a component in different receptor complexes that trigger symbiosis or immune responses. The possibility of complex formation between the ECDs of *Ps*LYK9-ECD and predicted co-receptors *Ps*LYR4-ECD and *Ps*LYR3-ECD was determined in a co-immunoprecipitation assay. As a control, the interaction between *Ps*LYK9-ECD and *Ps*SYM10-ECD, involved in Nod factor recognition in peas, was also estimated. To verify such interactions, the ECDs of LysM-RLKs *Ps*LYK9, *Ps*LYR4, *Ps*LYR3 and *Ps*SYM10 were synthesized in *E. coli* C41 cells. After incubation of *Ps*LYR4-ECD or *Ps*LYR3-ECD carrying the 6xHIS tag with *Ps*LYK9-ECD carrying the FLAG tag followed by purification on the column with anti-6xHIS antibodies, we observed simultaneous elution of *Ps*LYR4-ECD and *Ps*LYK9-ECD, as well as *Ps*LYR3-ECD and *Ps*LYK9-ECD (Figure 4). Therefore, complex formation between *Ps*LYK9 and both pea co-receptors is possible. In contrast, a weak interaction between *Ps*LYK9-ECD and *Ps*SYM10-ECD was observed (Figure 4).

### 2.5. Synthesis of the PsLYK9-ECD, PsLYR4-ECD and PsLYR3-ECD ECDs in Insect Cells

**Figure 5:**
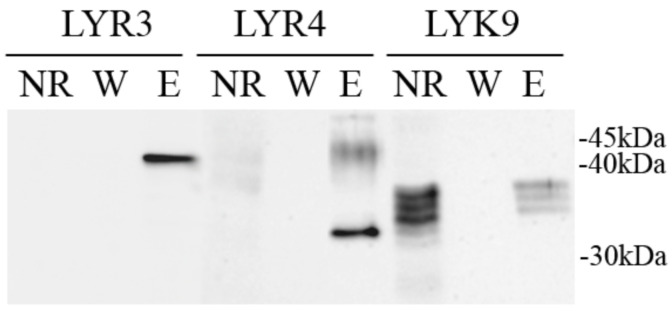
Purification of the *Ps*LYK9-ECD, *Ps*LYR4-ECD and *Ps*LYR3-ECD extracellular domains from insect cells using µMACS columns containing the MicroBeads with immobilized anti-HIS antibodies. Elution was carried out with triethylamine. NR—non-retained fraction, W—washing, E—elution.

Fragments of *Lyk*9, *Lyr4* and *Lyr3* genes encoding the putative ECDs of these LysM-RLKs were amplified using cDNA as a matrix and inserted into a pFastBac1 vector in frame with a sequence encoding the 6xHis tag. After heterologous expression, performed in Sf9 insect cells, recombinant *Ps*LYK9-ECD, *Ps*LYR4-ECD and *Ps*LYR3-ECD proteins accumulated in the soluble cell fraction but not in the culture medium (Figure 5). Western blot analysis showed that the molecular weights of *Ps*LYK9-ECD, *Ps*LYR4-ECD and *Ps*LYR3-ECD were approximately 35, 32 and 42 kDa, respectively (Figure 5). These weights were higher than those predicted (23 kDa for *Ps*LYK9-ECD, 27 kDa for *Ps*LYR4-ECD and 24 kDa for *Ps*LYR3-ECD), probably owing to protein glycosylation in Sf9 insect cells. The stability of *Ps*LYK9-ECD, *Ps*LYR4-ECD and *Ps*LYR3-ECD was tested using the Tycho NT.6 system.

### 2.6. Analysis of Receptor Binding with Ligands Using Thermophoresis

To verify the role of *Ps*LYK9-ECD, *Ps*LYR4-ECD and *Ps*LYR3-ECD as receptors, the analysis of their binding with ligand was performed. Binding experiments for purified His-tagged *Ps*LYK9-ECD, *Ps*LYR4-ECD and *Ps*LYR3-ECD ECDs with different chitin (CO4 and CO5) and chitosan oligomers (CO5-DA–CO8-DA) were performed using microscale thermophoresis. *Ps*LYK9-ECD, *Ps*LYR4-ECD and *Ps*LYR3-ECD were expressed in insect cells and purified on µMACS MicroBeads conjugated to anti-His monoclonal antibodies. To determine their affinities toward ligands, the proteins were fluorescently labeled and binding was measured with increasing concentrations of different chitin and chitosan oligomers. The equilibrium dissociation constant (Kd) values were calculated from the binding curves and are presented in Table 2.

The Kd values show that the affinity of *Ps*LYK9-ECD for chitotetraose (CO4) and chitopentaose (CO5) was in the low micromolar range (Table 2). These direct binding measurements suggest that the *Ps*LYK9 is involved in the perception of CO4-5, triggering symbiotic development with AM fungi. This is in line with the results of the phenotypic analysis of *lyk9* mutants. In contrast, no binding with CO4-5 was observed for *Ps*LYR4-ECD. However, *Ps*LYK9-ECD and *Ps*LYR4-ECD showed direct binding with chitosan oligomers (CO5-DA–CO8-DA), inducing defense reactions in peas (Table 2, Supplementary Materials Figure S1). Slightly stronger binding was apparent with CO6-DA–CO8-DA for both receptors. The Kd values will be more precise in our future experiments on proteins extracted from large volumes of cultured insect cells.

The binding affinity of *Ps*LYR3 with short-chain COs was also tested in our experiments and there was no binding under the current experimental conditions (data not shown).

### 2.7. Molecular Modeling

The aim of the in-silico experiment was to investigate the mode of binding between *Ps*LYK9 and CO5 and CO8-DA ligands. Homology modeling was performed using the SWISS modeler to obtain the structure of the *Ps*LYK9 ECD (*Ps*LYK9-ECD) using the 5LS2 (the Protein Data Bank; PDB code) LysM-type receptor kinase (*Lj*LYS6 = *Lj*CERK6) as a template. Searching of binding sites in *Ps*LYK9-ECD revealed five possible places (1–5) for binding (Supplementary Materials Figure S2). We used Glide to generate the receptor grid for each site and perform docking between *Ps*LYK9-ECD and CO5 and CO8-DA. Flexible extra-precision (XP) docking was performed without any constraints. In these experiments, binding affinity was observed in site 5 for both ligands; however, as this site is situated near the C- and N-terminus, this is unlikely to occur in a real cell (as these parts will be in a membrane and the protein tertiary structure would be changed). Therefore, we decided to ignore the fifth site because of its location.

**Figure 6:**
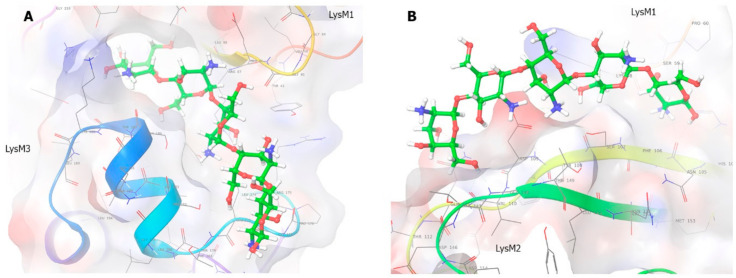
Molecular modeling of *Ps*LYK9-ECD binding with CO5. (**A**) Binding site 1: This figure shows the computed bonds between *Ps*LYK9-ECD and CO5 at site 1. CO5 binds *Ps*LYK9-ECD at site 1 along the groove above the LysM3 domain and partly in the cavity between LysM1 and LysM3. (**B**) Binding site 2: The figure shows the complex-forming bonds at site 2 for a pose with the best score. The complex at site 2 is formed by the protein and the ligand above the groove in the LysM2 and in the cavity between the LysM1 and LysM2 domains. Ligand is attached outwardly at site 2; thus, CO5 is exposed to the solvent more than at site 1.

The binding of CO5 and *Ps*LYK9 was tested for each of the 1–4 binding sites. The best GScore metric based on the binding affinity was observed for the complex at site 1 (Figure 6A), whereas the minimal energy of the model (combination of Coulomb-vdW energy, affinity and internal strain energy) was predicted for the complex at site 2 (Figure 6B). Ligand binding with a protein at site 2 was similar (but not identical) to chitin binding with *At*CERK1-ECD and *Lj*LYS6 from *L. japonicus* [14,17].

According to the model, CO5 binds *Ps*LYK9 at site 1 along the groove above the LysM3 domain and partly in the cavity between LysM1 and LysM3 domains (Figure 6A, Supplementary Materials Figure S3). This binding is characterized by many non-covalent bonds and good affinity; however, some corrupted (bad) bonds (which are not possible for the right structure of the protein) occur. The complex at site 1 has eight H-bonds, three salt bridges and three bad bonds (good: Ser186, Asn74, Glu178, Gly85, Gly177; bad: Asn89, Arg87, Met153). Viewing different possible conformations for this site, the ligand fluctuates significantly. However, the first three best conformations are situated at similar positions, showing the best position for binding at this site.

Although binding at site 2 does not seem as strong as that at site 1, it demonstrates the minimal energy of the model (Figure 6B, Supplementary Materials Figure S4). The complex is formed by the protein and the ligand located above the groove in the LysM2 domain and partly between the LysM1 and LysM2 domains. The upper part of the ligand varies a little among the different conformations but the mechanism of binding is the same. The ligand is attached facing outward; thus, CO5 is exposed to the solvent more than at site 1. The pose with the best GScore suggests that binding is characterized by six H-bonds, one salt bridge and three bad bonds (good: Gly55, Asp109, Gln111; bad: Thr149). The localization of P145 is not directly linked with the binding site of CO5 in *Ps*LYK9 (Supplementary Materials Figure S5), which probably reflects the weak impact of P145L replacement in *lyk9-3* mutants on symbiotic development with AM fungi.

**Figure 7:**
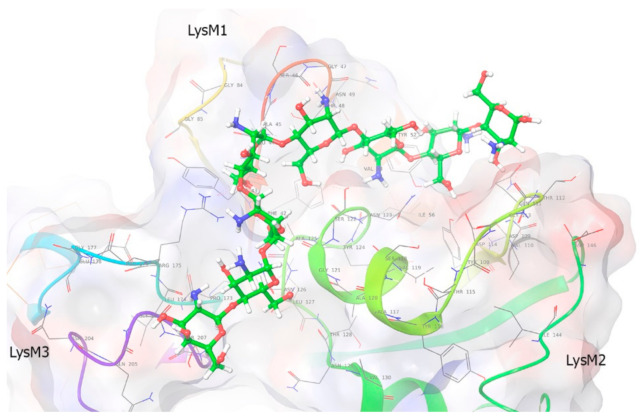
Molecular modeling of PsLYK9-ECD binding with CO8-DA. The main ligand conformation pattern is partly placed at the groove between LysM1 and LisM2 (four upper ligand rings), approaching LysM2, whereas the rest of the ligand descends to the central groove between LysM3 and LysM2. The type of binding is partly hydrophobic (at the area of LysM2 domain) and partly polar at the side of binding between LysM2 and LysM3. The lower and upper CO8-DA molecular rings are half-available to the solvent.

We also docked *Ps*LYK9-ECD and deacetylated CO8 (CO8-DA) to investigate the influence of the degree of polymerization and acetylation on binding (Figure 7, Supplementary Materials Figure S6). CO8-DA binds *Ps*LYK9-ECD only at site 2. Therefore, the binding was better, with an increasing degree of polymerization for CO molecules. A similar tendency has been described for *At*CERK1-ECD from *Arabidopsis* [23]. CO8-DA binding affinity and the model energy for the complex at site 2 are much higher than those for the previous complex with CO5. The best pose has a GScore = −12.611, Emodel = −90.371. Hence, longer molecules may be more efficient in the activation of the receptor complex. Binding is characterized by 8 H-bonds, 1 aromatic bond and 10 bad bonds (good: Ser46, Thr112, Asp114, Gly121, Ser122, Ala125, Asn126, Gly206; bad: Thr112, Asp114, Ser122, Asn123, Arg175). The ligand with the best scoring function value is partly placed at the interdomain groove between LysM1 and LysM2 (four upper ligand rings), approaching LysM2, whereas the rest of the ligand descends to the central groove between LysM3 and LysM2.

To conclude, binding site 2 is the universal site for binding as different ligands bind here, although one more binding site was observed for CO5 in *Ps*LYK9. The analysis showed that binding activity increased with an increasing degree of polymerization. Additionally, deacetylated CO8 binds better than acetylated CO8; thus, acetylation influences binding negatively.

## 3. Discussion

The viability of plants depends on their capacity to respond quickly to variable signals produced by soil microbiota. Chitin and chitosan derivatives such as their oligomers play an essential role in plant interactions with phytopathogenic and AM fungi. Here, the ability of pea plants to effectively distinguish these compounds was shown to be related to the LysM-RLKs *Ps*LYK9, probably as a component in receptor complexes triggering different signaling pathways. Plant response to COs depends on the length and degree of acetylation of these compounds. Analysis of pea mutants impaired in the *lyk9* gene showed that LysM-RLK *Ps*LYK9 may regulate the pea plant responses to COs with differing degrees of polymerization and acetylation, resulting in a decreased expression level of marker genes. Analysis of its binding capacity showed that *Ps*LYK9 and its possible co-receptor *Ps*LYR4, synthesized as extracellular domains in insect cells, could bind deacetylated oligomers CO5-DA, CO6-DA, CO7-DA and CO8-DA at low micromolar concentrations. Moreover, stronger binding was apparent with CO8-DA. In addition, the *Ps*LYK9 and *Ps*LYR4 formed a complex, as was shown in the co-immunoprecipitation assay. Similarly, the complex between the highly homologous proteins *Mt*LYK9 and *Mt*LYR4 was found to induce an immune response in *M. truncatula* plants [11,14]. This suggests that the receptor complex consisting of *Ps*LYK9 and *Ps*LYR4 is able to trigger a signal pathway that stimulates the immune response in pea plants. Indeed, long-chain chitosan oligomers are effective elicitors of defense reactions in pea plants. Deacetylated oligomers such as heptamers and octamers (CO7-DA, CO8-DA) were the most effective elicitors inducing the accumulation of phytoalexins and synthesis of pathogenesis-related proteins in pea, whereas CO5-DA and CO6-DA showed effects at significantly higher concentrations [31,32,33,34]. As a result, treating pea plants with these compounds significantly stimulated the resistance to phytopathogenic fungi. Previously, we showed that reduction of *PsLYK9* gene expression led to increased susceptibility to infection with phytopathogenic fungi [16]. This finding agrees with current data about *Ps*LYK9’ capacity to respond to deacetylated oligomers released into the medium during pea plants’ interaction with phytopathogenic fungi.

At the same time, the *Ps*LYK9 may be a component of another receptor complex that regulates symbiosis development between pea plants and AM fungi. In accordance with this, the *lyk9-1* and *lyk9-2* mutants demonstrated decreased levels of colonization by mycorrhizal fungi and reduced response to CO5 treatment in our experiments. Moreover, binding of CO4 and CO5 by *Ps*LYK9 was demonstrated using microscale thermophoresis. Similarly, the homolog of this receptor in *M. truncatula*, the *Mt*LYK9, was able to bind CO4 and CO5 as it was found recently [11]. Modeling showed that CO5 may be involved in binding with two potential sites in *Ps*LYK9. One of them is similar to a typical site for binding of chitin and chitin oligomers in the LysM2 domain previously found in *At*CERK1-ECD and *Lj*LYS6 from *L. japonicus* [14,17]. At the same time, the importance of amino acid residues in the LysM1 domain for binding with this ligand was also shown in binding site 2. Predicting another binding site in *Ps*LYK9 for CO5 as well as the importance of other LysM domains in binding with ligand may highlight the specific features of CERK1-like receptors in legume plants. Future experiments based on site-directed mutagenesis should be performed to verify this hypothesis. To find a possible co-receptor of *Ps*LYK9 in the binding of CO4 and CO5, we tested its capacity to form complexes with other pea LysM-RLKs. Based on the complex formation between *Ps*LYK9 and *Ps*LYR3, we suggested that *Ps*LYR3 may be a possible co-receptor involved in binding with low molecular weight CO4 and CO5. However, the binding affinity of *Ps*LYR3 with short-chain COs was tested in our experiments and showed no essential binding under current experimental conditions. This suggests that other co-receptors for *Ps*LYK9 essential for binding with CO4-5 should be found in the future.

## 4. Materials and Methods

### 4.1. Plants and Microorganisms

The wild-type *Pisum sativum* L. cultivars Frisson and Finale were used in this study. The plant *Plecthrantus australis* was used for obtaining of mycorrhizal fungi inoculum. The arbuscular mycorrhiza fungi *Rhizophagus irregularis* DAOM 197198 from Agronutrition company (Labege, France) was used as a plant inoculum. Bacterial strain *E. coli* C41 was used for protein synthesis and XL1-Blue MRF’, DH5α strains were applied for routine transformation procedures (Stratagene, CA, USA). *E. coli* strains were cultured in LB liquid medium [35] on an orbital shaker (Heidolph Unimax, Schwabach, Germany) at 37 °C in the presence of the antibiotics.

### 4.2. Plant Growth Conditions

Seeds of *P. sativum* were surface-sterilized for 10 min with concentrated sulfuric acid, rinsed with sterile deionized water five times and then germinated for 4–5 days at 22 °C on 1% aqueous agar in Petri dishes in the dark. After germination (for 4–5 days), seedlings were transferred into pots with vermiculite or mineral substrate and grown in a growth chamber at 21 °C in a 16 h/8 h light/dark cycle at 60% humidity.

### 4.3. Targeting-Induced Local Lesions in Genomes (TILLING) Screens

The analysis was performed using the TILLING approach, which relied on the construction of high-quality pea mutant collection (cv. Cameor) [30] (https://www6.dijon.inra.fr/umragroecologie_eng/Research-Cluster/GEAPSI) available at UMR Agroécologie, INRA, France, followed by screening of mutants (IPS2, INRA, Orsay, France). Searching for mutations was performed using the next-generation sequencing technology to detect putative mutations within two amplicons of the *Lyk9* gene. Prediction of the amino acid changes that affect protein function was made using the program [36] (sift.jcvi.org). In total, 6 mutant lines were available for work: 3693, 4249, 4542, 3562, 3079 and 3631 (Supplementary Materials Table S1). Mutant lines were evaluated in M4 and M5 generations for *Lyk9* gene homozygosity and 3 lines were used for phenotypic analysis in detail: 3631 (*lyk9-1*), 3079 (*lyk9-2*) and 4249 (*lyk9-3*). Parental pea cultivar Cameor was used as a control.

### 4.4. Fungal Inoculum Preparation

The roots of *Plecthrantus australis* plants infected with *Rhizophagus irregularis* DAOM 197198 were used for inoculum preparation after at least 1–3 months of co-cultivation. *P. australis* roots colonized with *R. irregularis* were separated from the growth substrate and cut into pieces of about 1 cm, then thoroughly mixed. The resulting root mass was mixed (1:1) again with the growth substrate and divided into equal parts per every pot (about 1 g).

### 4.5. Plant Inoculation with Rhizophagus Irregularis

Plant seedlings were placed in 250 mL plastic vessels with a mineral substrate (150 g), which is silica-rich marl (Krasnodar, Russia), supplemented with 1 g/L calcium orthophosphate. Vessels and substrate were separately sterilized by autoclaving for 30 min at 134 °C and 0.22 MPa. Seedlings were placed in the substrate, and the inoculum (about 1 g) was placed at the bottom of each dimple (1 seedling per vessel). Plants were fed once a week with modified Hoagland’s solution without phosphate (25 mL per vessel) [37,38] and watered as needed.

The parameters of mycorrhization and expression of AM symbiosis-specific genes were assessed 4 weeks after inoculation. For gene expression analysis, the plants were removed from the substrate and their root systems were thoroughly washed. About 100 mg of lateral root fragments were collected individually from each plant in 1.5 mL Eppendorf tubes and were immediately frozen at −80 °C. In total 5–6 plants in each variant were taken for analysis. For arbuscular mycorrhiza analysis, fragments of lateral roots were collected individually from each plant in 1.5 mL Eppendorf tubes and were stored at −20 °C.

### 4.6. Analysis of Mycorrhization

Sheaffer Black Ink staining was performed to visualize fungal structures in the root samples [39]. Root fragments having a total length of 30 cm for each plant (n = 6) were mounted on glass slides in glycerol. The AM development was examined using the Axiovert 35 light microscope (Zeiss/Opton, Oberkochen, Germany) and quantitatively assessed according to Trouvelot et al. [40] by the following parameters: *F%*—frequency of fungus in root fragments, *M%*—intensity of intraradical mycelium development (reflects the proportion of the root length colonized by the fungus) and *a*%—arbuscule abundance in mycorrhizal root fragments (characterizes the functional state of the fungus). For statistical analysis, the parameters were subjected to arcsine transformation to normalize the data [41] and compared by one-way analysis of variance (ANOVA) (SPSS 12.0 package, SPSS Inc., Chicago, IL, USA).

### 4.7. Treatment of Pea Seedlings with COs

Four- to five-day-old seedlings of cv. Cameor and *lyk9* mutant lines were transferred in glass jars with 10^−5^ M water solutions and incubated for 24 h. Fully acetylated CO5 (Megazyme, Wicklow, Ireland) and deacetylated COs with main degree of polymerization around eight (CO8-DA): Mn = 1089, Mw = 1514, Ip = 1.39, CDS = 93% in Cl-form (The Center of Bioengineering Russian Academy of Science, Moscow, Russia), were used for treatment. After treatment, the pea root fragments corresponding to responsive zones were harvested for RNA extraction and gene expression analysis and immediately frozen at −80 °C.

To test the binding capacity of *Ps*LYK9-ECD, *Ps*LYR4-ECD expressed in insect cells, the chitin oligomers like chitotetraose (CO4) (Megazyme, Wicklow, Ireland) and chitopentaose (CO5) (Megazyme, Ireland) were applied. The chitosan oligomers CO5-DA (Seikagaku, Tokyo, Japan), CO6-DA (Carbosynth, Compton, UK), CO7-DA (Carbosynth, UK) and CO8-DA (Carbosynth, UK) were also used.

### 4.8. Molecular Cloning

The fragments of *P. sativium* L. *Lyk9*, *Lyr4* and *Lyr3* genes encoding the extracellular domains of corresponding proteins were amplified by Polymerase Chain Reaction (PCR) using cDNA of cv. Finale with Phusion Flash High-Fidelity PCR Master Mix (Thermo Fisher Scientific, Waltham, MA, USA) and primers containing restriction sites.

The PCR products corresponding to coding sequences from 91 to 705 bp for the *Lyk9* (615 bp), from 22 to 771 bp for *Lyr4* (750 bp) and from 70 to 728 for *Lyr3* (658 bp) were gel-purified and inserted into the baculovirus expression vector pFastBac1 (Thermo Fisher Scientific, USA) using *XhoI*/*HindIII* (*Lyk9*) and *BamHI/EcoRI* (*Lyr4* and *Lyr3*) restriction sites and sequenced using M13 forward and reverse primers. Using SignalP-5.0 server, the signal peptides in the proteins were predicted and transmembrane domains were found by means of TMHMM Server, v. 2.0. The vector contained the sequence encoding GP67 (envelope surface glycoprotein 67) for protein expression in insect cells. For cloning of *Lyr3,* the sequence encoding GP67 was removed from the construct in pFastBac1. At the next step, the fragments were re-cloned into the bacmid in *E. coli* DH10Bac cells (Bac-to-Bac expression system, Thermo Fisher Scientific).

### 4.9. Bacmid Isolation and Purification

DH10Bac cells with bacmids containing the constructs with genes of interest were grown for two days. After precipitation, the cell culture was resuspended in 20 mM Tris (pH 8.0), 200 mM NaOH and 1% sodium lauroyl sarcosine. After 5 min of incubation at room temperature, 3 M potassium acetate (pH 5.2) was added and cooled to −20 °C. Then, it was precipitated with isopropanol and resuspended with 10 mM Tris (pH 8.0), 1 mM ethylenediaminetetraacetic acid (EDTA) and in the presence of RNAse A (10 mg/mL). The bacmids were washed with phenol:chloroform (in a ratio of 1:1) followed by washing with 80% ethanol under sterile conditions.

### 4.10. Heterologous Synthesis of PsLYK9-ECD, PsLYR4-ECD and PsLYR3-ECD Extracellular Domains in Sf9 Insect Cell

The Sf9 cell line was obtained from European Collection of Authenticated Cell Cultures (ECACC) General collection (ECACC 89070101). Adhesive cell culture was grown in Sf900III serum-free medium (SFM) (Thermo Fisher Scientific, USA) and was routinely maintained according to the manufacturer’s instructions in T25 cultural flasks (Eppendorf, Germany) at 27 °C. The Bac-to-Bac baculovirus expression system (Invitrogen, Waltham, MA, USA) was used to gain recombinant proteins in insect cell culture.

Bacmid transfection was performed with 2 µg of plasmid DNA mixed with 8 µL of Cellfectin II liposomal reagent, kept for 15 min at room temperature until liposomes were formed, and added to 8 × 10^5^ cells in a well of a 6-well plate. After 6 days, the virion-containing culture fluid was used to produce the next generation of recombinant baculoviruses with an increased titer of viral particles. The *Ps*LYK9-ECD and *Ps*LYR4-ECD proteins were synthesized using the fourth generations of baculoviruses. The *Ps*LYR3-ECD expression was carried out using the third generation. For this, 100 µL of a suspension of viral particles was added to 2 × 10^6^ Sf9 cells in a well of a 6-well plate. After 96 h of expression, the cells were harvested and separated by centrifugation from the culture medium. Protein samples were prepared and analyzed by immunoblotting with 6xHis-tag antibodies.

### 4.11. Co-Immunoprecipitation Assay

Co-immunoprecipitation was carried out using a µMACS kit (Miltenyi Biotec, Bergisch Gladbach, Germany) containing the MicroBeads with immobilized anti-His and anti-DYKDDDDK (also known as FLAG-tagged) antibodies. The pellet of *E. coli* cells was resuspended in lysis buffer (50 mM tris-HCl pH 8.0, 1% Triton X-100, 150 mM NaCl) and cell lysate was obtained via the sonication (3 × 20 s). These lysates containing His-tagged (*Ps*LYR3-ECD, *Ps*LYR4-ECD and *Ps*SYM10-ECD) or FLAG-tagged (*Ps*LYK9-ECD) proteins were co-incubated with MicroBeads for 1 h on ice and then were loaded onto a µMACS column placed in the magnetic field of a µMACS separator. Then, column with associated proteins was washed with lysis buffer two times. After elution with denaturing elution buffer, the precipitated proteins were analyzed by sodium dodecyl sulfate polyacrylamide gel (SDS-PAGE). In our investigation, we used two approaches to study co-immunoprecipitation using a µMACS kit. In the first variant, we passed FLAG-tagged proteins and His-tagged proteins after co-incubation through magnetic MicroBeads with anti-His antibodies. In other variant, His-tagged and FLAG-tagged proteins were passed through the column with MicroBeads with immobilized anti-FLAG antibodies.

### 4.12. Microscale Thermophoresis

Binding experiments were performed by microscale thermophoresis with a Monolith NT.115 (NanoTemper Technologies, Munchen, Germany). Purified *Ps*LYK9-ECD, *Ps*LYR4-ECD and *Ps*LYR3-ECD proteins were labelled with the Monolith NT Protein Labeling kit RED-tris-NTA according to the instructions provided by the manufacturer, using a 1:1 protein:dye molar ratio. For binding experiments, the 20 nM labelled protein was incubated with a range of ligand concentrations made by serial dilutions, in 50 mM Tris buffer pH 7.4, 10 mM MgCl_2_, 150 mM NaCl, 0.05% Tween 20 at room temperature for 30 min. Standard treated capillaries (NanoTemper Technologies, Munchen, Germany) were loaded and the measurements were performed at 25 °C, 20% LED power and 40% microscale thermophoresis power, 20 s laser-on time, 1 s laser-off time.

### 4.13. RNA Isolation and cDNA Synthesis

Pea roots were harvested and frozen in liquid nitrogen. Total RNA was isolated using about 50–100 mg tissue per sample. Samples were thoroughly ground in a mortar to a fine powder in liquid nitrogen, at least three biological replicates per each condition. RNA was extracted using PureZol reagent (BioRad laboratories, Hercules, CA, USA). After a DNase (Thermo Fisher Scientific, Waltham, MA, USA) treatment, the samples were extracted with an equal volume of chloroform, and RNA was precipitated from the aqueous phase with 3 M sodium acetate and ethanol and subsequently quantified with a spectrophotometer UV-1280 (Shimadsu, Kyoto, Japan). RNA purity was checked by measuring spectrophotometric ratios of A260/A280. The efficacy of the DNase treatment was checked by using controls without reverse transcriptase for subsequent quantitative reverse transcription PCR (qPCR) analysis. RNA (from 1 to 2.5 g) was used for cDNA synthesis with RevertAid Reverse Transcriptase (Thermo Fisher Scientific) for 1 h at 42 °C followed by heating to 95 °C, for 5 min. Aliquots of the cDNA were diluted 1:10 for qPCR analysis.

### 4.14. Quantitative Reverse Transcription Polymerase Chain Reaction (qRT-PCR)

The qRT-PCR analysis was performed on a CFX-96 real-time PCR detection system with a C1000 thermal cycler (Bio-Rad Laboratories, Richmond, CA, USA), and SYBR Green intercalating dye was used for detection (Bio-Rad Laboratories, Richmond, CA, USA). Each PCR reaction was carried out in a total volume of 10 µL. The following PCR program was used: 40 cycles of 95 °C for 30 s, 54 °C for 30 s, 72 °C for 40 s. All reactions were performed in triplicate and averaged. Cycle threshold (Ct) values were obtained with the accompanying software and data were analyzed with the 2^−ΔΔCt^ method [42]. The relative expression was normalized against the constitutively expressed *Ubiquitin* and *Actin* genes in pea. All the primers used in the expression analysis are listed in Supplementary Materials Table S2. All primer pairs were designed using the Vector NTI program and produced by Evrogen Company (Moscow, Russia) (www.evrogen.com). Each experiment was repeated at least three times with independent biological samples.

### 4.15. Molecular Modeling

The homology modeling was performed with the SWISS tool (https://swissmodel.expasy.org/) to obtain the structure of *Ps*LYK9 extracellular domain (*Ps*LYK9-ECD). Fifty alignment templates for *Ps*LYK9-ECD amino acid sequence were obtained. One of them (based on 5LS2 LysM-type receptor kinase *Lj*LYS6) was chosen for further modelling as it had the best sequence similarity and good alignment metrics, GMQE (0.86) and QMEAN (0.45) (Supplementary Materials Table S3). Next, the protein heteroatom states were generated with Epik module under pH 7.0 ± 2.0, the optimization was done with PROPKA at pH 7.0, and finally, protein was minimized with OPLS3e force field.

The modeling of CO5 and CO8-DA ligands was done using the Schrodinger two-dimensional (2D) Sketcher and further transformation to three-dimensional (3D) space in the program. Both ligands were prepared with “LigPrep” tool. The library consisting of 100 conformations was created for each of them (OPLS3e force field, pH 7.0 ± 2.0, Epik, desalt as done, tautomers were included).

### 4.16. Statistical Methods and Computer Software

The expression levels of the gene of interest (GOI) relative to the reference genes *Ubiquitin* and *Actin* were calculated for each cDNA sample using the CFX Manager software version 2.1 (BioRad Laboratories, Richmond, CA, USA). The expression levels of GOI were calculated as ratio of treated samples to control samples. Statistical analysis was conducted by SIGMAPLOT 13. Multiple alignment of nucleotide sequences was performed using Clustal W [43] using Vector NTI Advance 10 (InforMax, http://www.informaxinc.com). One-way ANOVA was used to compare gene expression levels in treated pea roots.

## Figures and Tables

**Table 1:** Mutations in the *lyk9* gene and their effect in mutant plants.

Mutant Line	Mutation	DNA Position	Protein Position	Localization
3631 (*lyk9-1*)	G→A	4533	G485R	kinase domain, the activation loop
3079 (*lyk9-2*)	C→T	4340	L470F	kinase domain, the activation loop
4249 (*lyk9-3*)	C→T	512	P145L	LysM2 motif of extracellular domain

**Table 2:** The equilibrium dissociation constants of *Ps*LYK9-ECD and *Ps*LYR4-ECD with COs.

Protein	Ligand	Kd, µM (±SD)
LYR4-ECD	CO4	-
	CO5	-
	CO5-DA	45 ± 20
	CO6-DA	22 ± 11
	CO7-DA	19 ± 13
	CO8-DA	8 ± 5
LYK9-ECD	CO4	2.1 ± 0.6
	CO5	5 ± 1.9
	CO5-DA	76 ± 25
	CO6-DA	42 ± 23
	CO7-DA	31 ± 11
	CO8-DA	26 ± 15

“-“—no binding was found; SD—standard deviation.

## Data Availability

Not applicable.

## Supplementary Materials

The following are available online at https://www.mdpi.com/1422-0067/22/2/711/s1, Figure S1: Analysis of *Ps*LYR4-ECD binding to different chitosan oligomers using microscale thermophoresis (MST), Figure S2: Searching of binding sites in the *Ps*LYK9-ECD, Figure S3: Diagram of *Ps*LYK9-ECD binding with CO5, Figure S4: Diagram of *Ps*LYK9-ECD binding with CO5, Figure S5: Localization of P145 in relation to binding area in *Ps*LYK9-ECD, Figure S6: Diagram of *Ps*LYK9-ECD binding with CO8-DA, Table S1: Mutant lines found in the TILLING collection and carrying a mutation in the *PsLyk9* gene, Table S2: List of primers used in this study, Table S3: Features of *Ps*LYK9-ECD’ binding sites.
