# Supplementary material for: LysM Receptor-Like Kinase LYK9 of Pisum Sativum L. May Regulate Plant Responses to Chitooligosaccharides Differing in Structure
Source: Int J Mol Sci. 2021 Jan 12;22(2):711. doi: 10.3390/ijms22020711 (PMC7828211; doi:10.3390/ijms22020711)
Supplement: Supplementary file 1 [file ijms-22-00711-s001.pdf]

## Supplemental Data

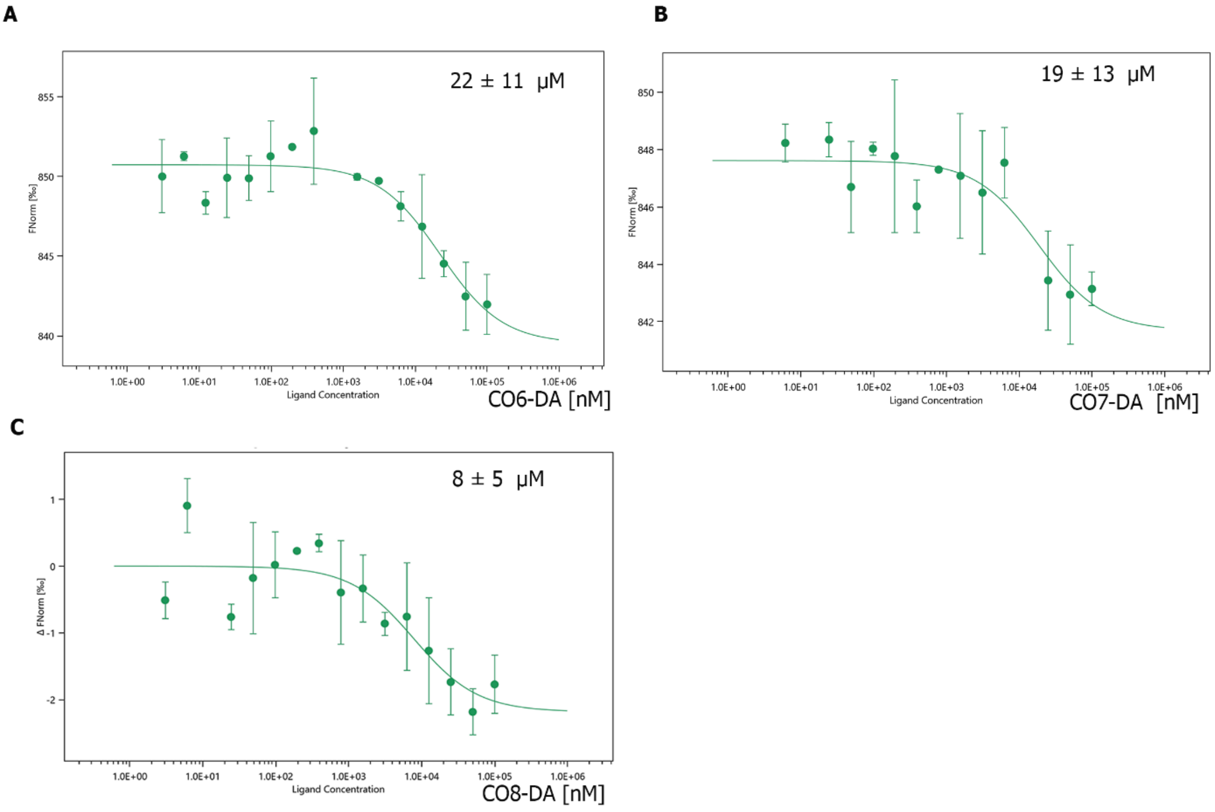


**Supplemental Figure S1**. Analysis of *Ps*LYR4-ECD binding to different chitosan oligomers using microscale thermophoresis (MST), (A) CO6-DA, (B) CO7-DA, (C) CO8-DA.

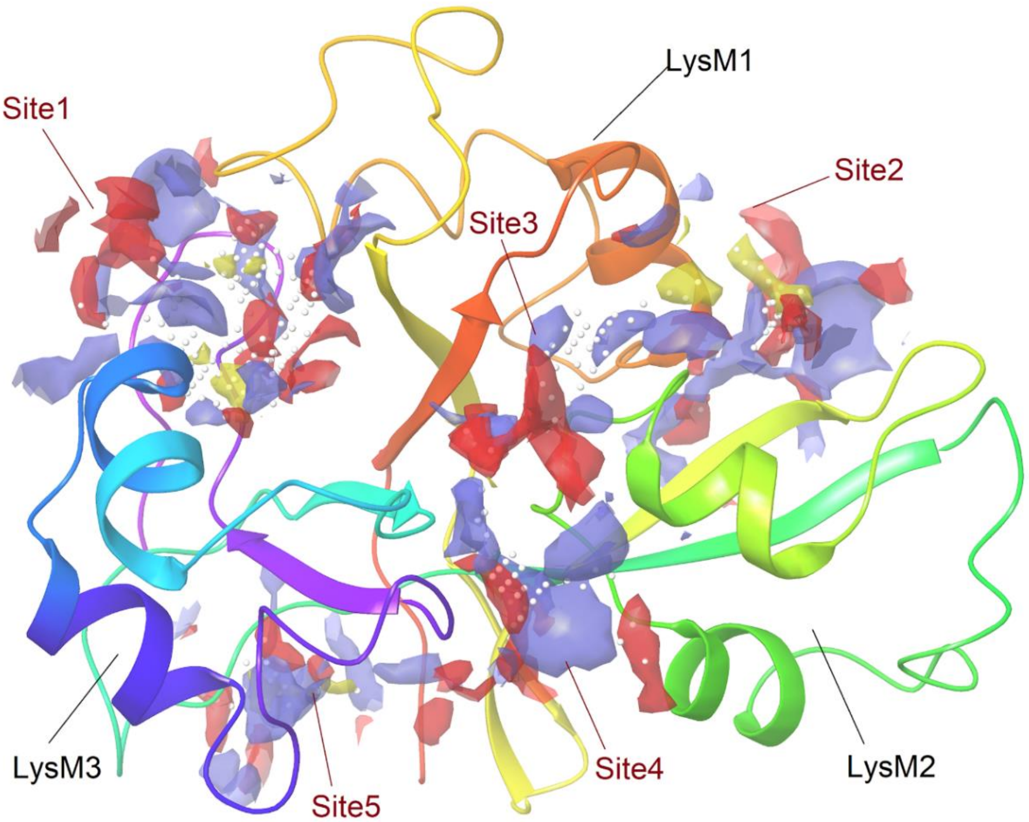


**Supplemental Figure S2**. Searching of binding sites in the PsLYK9-ECD revealed five potential places for binding. The binding sites are situated between LysM domains. Site 3 and site 4 are placed at the central groove, the 5 site occupies the area near the hypothetical transmembrane alpha helixes. Site 1 occupies the groove between LysM3 and LysM1 domains and site 2 is in the groove between LysM1 and LysM2.

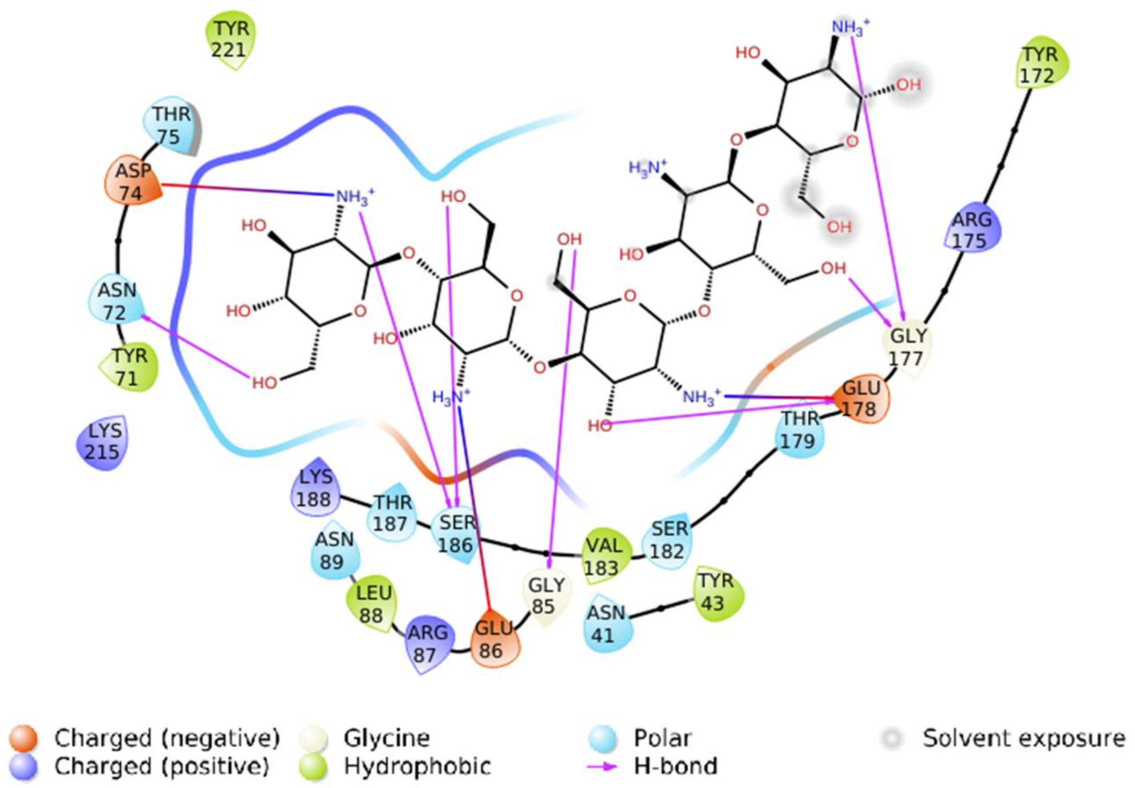


**Supplemental Figure S3.** Diagram of PsLYK9-ECD binding with CO5 (binding site 1). This binding is characterized by many non-covalent bonds and good affinity. The complex with best GScore and model energy for the site 1 has 8 H-bonds, 3 salt bridges and 3 “bad” bonds. “Good” bonds are marked at the diagram: Ser186, Asn74, Glu178, Gly85, Gly177. “Bad” bonds are not marked at the figure: Asn89, Arg87, Met153. It is well seen that CO5 interacts with polar and negative charged aminoasids and glycine. The outer part of the ligand surface is available to the solvent and is able to make bonds with water or hydrophilic liquids. The CO5 surface is charged in according to inner PsLYK9-ECD cavity surface.

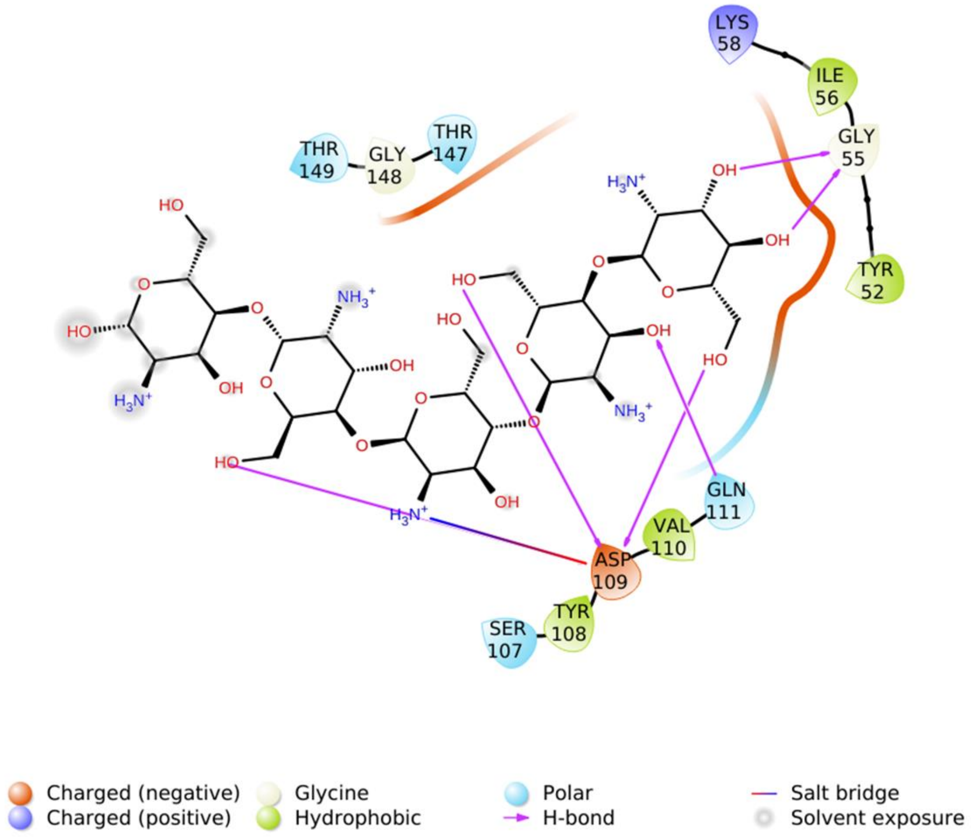


**Supplemental Figure S4**. Diagram of PsLYK9-ECD binding with CO5 (binding site 2). The ligand binds the protein near the groove but not inside it and characterized by 6 H-bonds, 1 salt bridge, 3 “bad” bonds. “Good” bonds are marked at the diagram: Gly55, Asp109, Gln111. “Bad” bonds are not marked at the figure: Thr149. The ligand interacts with polar, negatively and positively charged amino acids as well as with glycine and hydrophobic Val110. The CO5 surface is partly polar, negatively charged at the area of Thr149 and hydrophobic near the hydrophobic Pro60. Almost one-half of the ligand surface is available to the solvent.

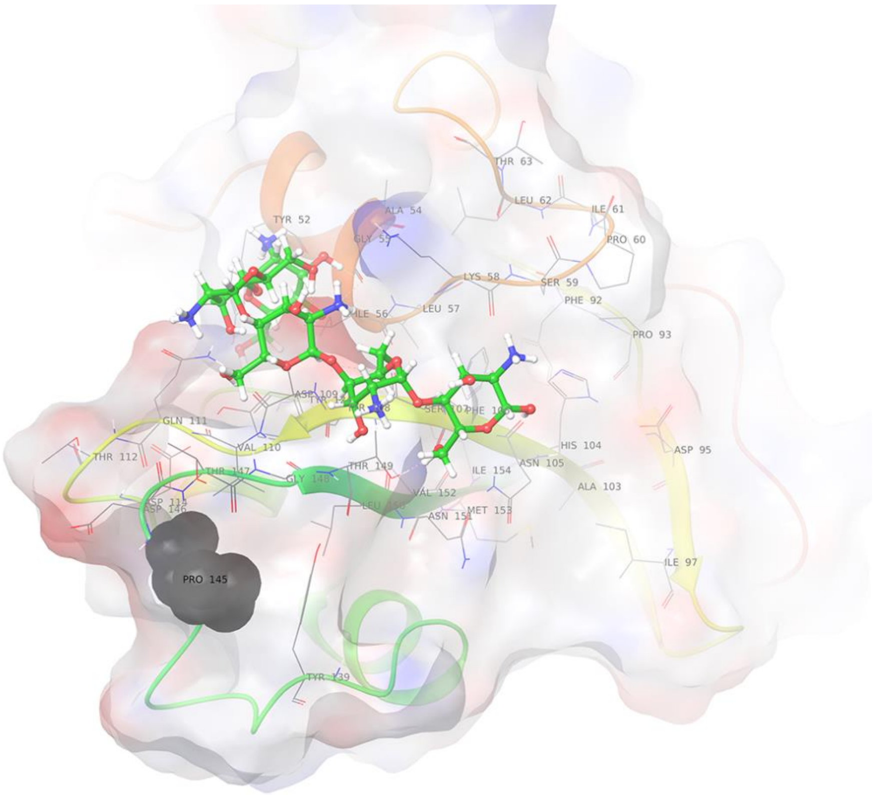


**Supplemental Figure S5**. Localization of P145 in relation to binding area in PsLYK9-ECD. P145 is situated at the LysM2 lower lateral loop under the binding site 2. However, it does not influence to the binding as it does not bind with CO5 or CO8-DA.

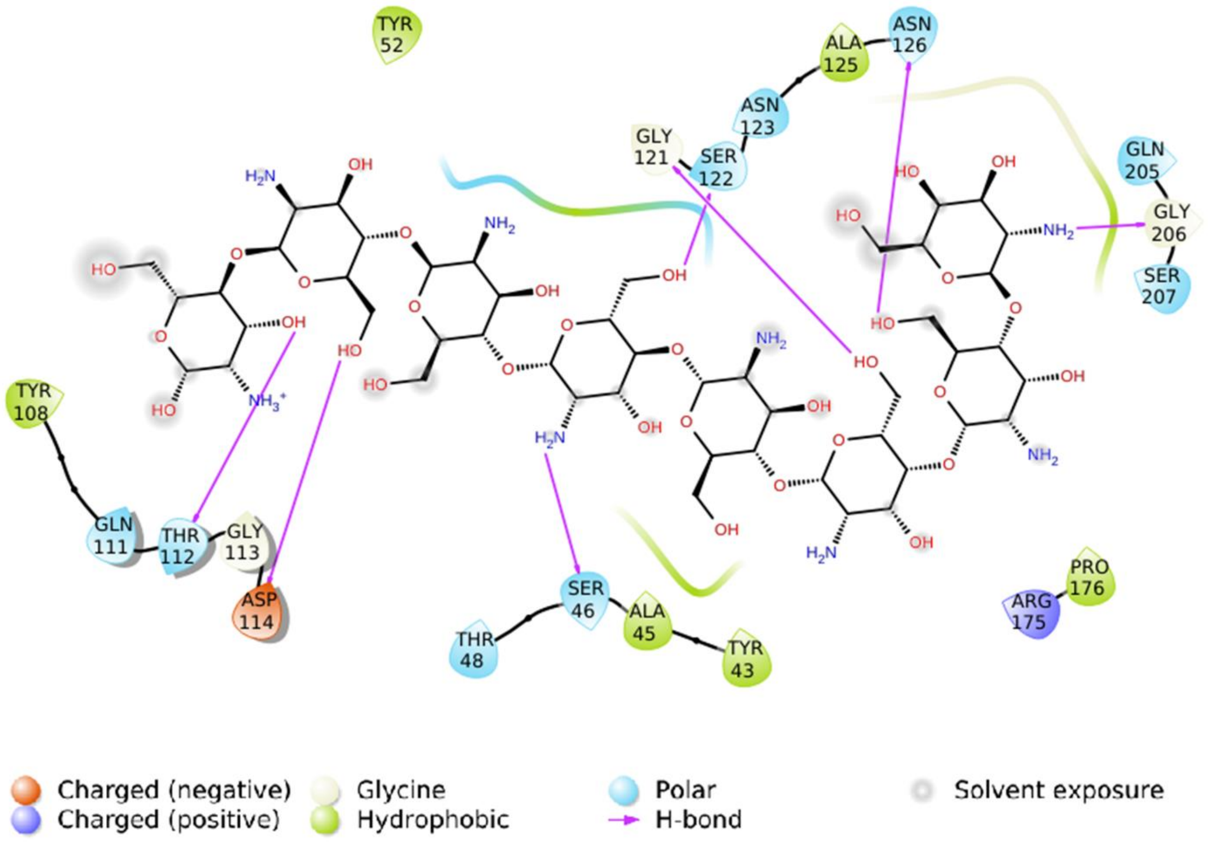


**Supplemental Figure S6**. Diagram of PsLYK9-ECD binding with CO8-DA. The binding is characterized by 8 H-bonds, 1 aromatic bond, 10 bad bonds. “Good” bonds are marked at the diagram: Ser46, Thr112, Asp114, Gly121, Ser122, Ala125, Asn126, Gly206. “Bad” bonds are not marked at the figure: Thr112, Asp114, Ser122, Asn123, Arg175. The complex is primarily formed with polar amino acids, with one negative charged amino acid and with glycines. The surface of the CO8-DA interacts with PsLYK9-ECD surface primarily in hydrophobic way. However, the ends of the molecule and some atoms along its “body” are available to the solvent.

**Supplemental Table S1**. Mutant lines found in the TILLING collection and carrying a mutation in the *PsLyk9* gene.

| # | Mutation | Families | Nature | AA Change | gDNA position | SIFT prediction | SIFT score | SIFT median sequence conservation | Sequence represented at this position |
|---|---|---|---|---|---|---|---|---|---|
| 1 | C→T | 3693 | MISSENSE | S122F | 443 | AFFECTED | 0,03 | 3,06 | 57 |
| 2 | C→T | 4249 | MISSENSE | P145L | 512 | AFFECTED | 0,01 | 3,02 | 60 |
| 3 | C→T | 4542 | MISSENSE | A184V | 629 | AFFECTED | 0 | 3,02 | 60 |
| 4 | G→A | 3562 | MISSENSE | A184T | 628 | AFFECTED | 0 | 3,02 | 60 |
| 5 | C→T | 3079 | MISSENSE | L470F | 4340 | AFFECTED | 0,02 | 3,01 | 64 |
| 6 | G→A | 3631 | MISSENSE | G485R | 4533 | AFFECTED | 0 | 3,01 | 63 |

**Supplemental Table S2**. List of primers used in this study.

| | |
|---|---|
| RT-PCR primers: | |
| *PE*_For | AGCTAGATCTTCTCTCGCCGTG |
| *PE*_Rev | CCACTCCCATCTTTAGCCACTAC |
| *MCA2*_For | TATTATCCGAGTGATGGCAA |
| *MCA2*_Rev | CTGCAACATTGACTGTTAGT |
| *PLC*_For | TCAACCTGCCATCAATGTTCTC |
| *PLC*_Rev | TTCTTTTGGACCATATCATCTACTGTC |
| *DELLA3*_For | GCAATATAGAATTAACCGCCACAAC |
| *DELLA3*_Rev | CGGATGAGCGGGACAACC |
| *WRKY33*_For | ACCCGAATTGTCCAACGAAGAA |
| *WRKY33*_Rev | TTAGCAGGAACAATCACAAGAGCA |
| *WRKY35*_For | GGGTTGTGAGTGTGAGGATTGG |
| *WRKY35*_Rev | TGGATGGTTGTGCGTTGAAGTA |
| *PUB22*_For | TGTCCGATCACAAAACAACAGC |
| *PUB22*_Rev | GAAGCTTCTTTGATGAGTTTCGC |
| *Ub*_For | ATGCAGATYTTTGTGAAGAC |
| *Ub*_Rev | ACCACCACGRAGACGGAG |
| *Actin*_For | CTCAGCACCTTCCAGCAGATGTG |
| *Actin*_Rev | CTTCTTATCCATGGCAACATAGTTC |
| | |
| Primers for protein expression in insect cells | |
| *Ps*LYK9-ECD_For | GG**CTCGAG**ATGTGCACAAAAGGTTGTTCATTAGCC (XhoI) |
| *Ps*LYK9-ECD_Rev | GG**AAGCTT**CGCTAATGATGATGATGATGATGAGCCCCACCCGC AAGACC (HindIII, stop and 6His tag) |
| *Ps*LYR4-ECD_For | GG**GGATCC**ACACTACTCATCCTCATAATCTCCA (BamH1) |
| *Ps*LYR4-ECD_Rev | GG**GAATTC**TCAATGATGATGATGATGATGACCACCACCCGCGG GAGGAGTTTGC (EcoRI, stop and 6His tag) |
| *Ps*LYR3-ECD_For | GG**GGATCC**CTAGGACAACAACCTTACATT (BamH1) |
| *Ps*LYR3-ECD_Rev | GG**GAATTC**TCAATGATGATGATGATGATGTTGAATTTGAGAAC TTTTAGGCTTATC (EcoRI, stop and 6His tag) |
| | |
| Primers for protein expression in *E. coli* cells | |
| *Ps*LYK9-ECD_For | CC**CTCGAG**TGCACAAAAGGTTGTTCATTAGCC (XhoI) |
| *Ps*LYK9-ECD_Rev | CC**AAGCTT**CGCTAAGCCCCACCCGCAAGAC (HindIII) |
| *Ps*LYR4-ECD_For | GG**GGATCC**GACACTAACCATGTTTTCTCTCATCT (BamHI) |
| *Ps*LYR4-ECD_Rev | GG**GAATTC**TCAACCACCACCCGCGGGAGGAGTTTGC (EcoRI) |
| *Ps*LYR3-ECD_For | GGGG**GGATCC**ACTAGGACAACAACCTTACATT (BamHI) |
| *Ps*LYR3-ECD_Rev | GGGG**GAATTC**TCATTGAATTTGAGAACTTTTAGGCTTATC (EcoRI) |

**Supplemental Table S3**. Features of PsLYK9-ECD' binding sites

The sites' metrics

| Site | SiteScore | Volume | Dscore | Size |
|---|---|---|---|---|
| 1 | 1.088 | 288.806 | 1.009 | 115 |
| 2 | 0.605 | 60.025 | 0.590 | 33 |
| 3 | 0.631 | 63.112 | 0.511 | 30 |
| 4 | 0.615 | 75.117 | 0.516 | 36 |
| 5 | 0.588 | 135.485 | 0.527 | 23 |

The best CO5 – PsLYK9-ECD poses according to XP GScore for each site

| Ligand | XP GScore | docking score | glide emodel |
|---|---|---|---|
| Ligand at site 1 | -10.214 | -9.082 | -57.424 |
| Ligand at site 2 | -9.795 | -8.663 | -75.486 |
| Ligand at site 3 | -9.802 | -8.670 | -59.802 |
| Ligand at site 4 | -9.151 | -8.019 | -59.665 |
| Ligand at site 5 | -11.274 | -10.142 | -77.548 |

The best CO8-DA – PsLYK9-ECD poses according to XP GScore for each site

| Ligand | XP GScore | docking score | glide emodel |
|---|---|---|---|
| Ligand at site 2 | -12.611 | -12.232 | -90.371 |
| Ligand at site 3 | -11.060 | -10.681 | -44.636 |
